# Interplay between HIV Entry and Transportin-SR2 Dependency

**DOI:** 10.1186/1742-4690-8-7

**Published:** 2011-01-30

**Authors:** Wannes Thys, Stéphanie De Houwer, Jonas Demeulemeester, Oliver Taltynov, Renée Vancraenenbroeck, Melanie Gérard, Jan De Rijck, Rik Gijsbers, Frauke Christ, Zeger Debyser

**Affiliations:** 1 Laboratory of Molecular Virology and Gene Therapy, Katholieke Universiteit Leuven, Kapucijnenvoer 33, VCTB+5, B-3000 Leuven, Flanders, Belgium; 2 Laboratory for Biomolecular Modelling, Katholieke Universiteit Leuven, B-3000 Leuven, Flanders, Belgium; 3 Laboratory of Biochemistry, Interdisciplinary Research Centre, Katholieke Universiteit Leuven-Kortrijk, B-8500 Kortrijk, Flanders, Belgium

## Abstract

**Background:**

Transportin-SR2 (TRN-SR2, TNPO3, transportin 3) was previously identified as an interaction partner of human immunodeficiency virus type 1 (HIV-1) integrase and functions as a nuclear import factor of HIV-1. A possible role of capsid in transportin-SR2-mediated nuclear import was recently suggested by the findings that a chimeric HIV virus, carrying the murine leukemia virus (MLV) capsid and matrix proteins, displayed a transportin-SR2 independent phenotype, and that the HIV-1 N74D capsid mutant proved insensitive to transportin-SR2 knockdown.

**Results:**

Our present analysis of viral specificity reveals that TRN-SR2 is not used to the same extent by all lentiviruses. The DNA flap does not determine the TRN-SR2 requirement of HIV-1. We corroborate the TRN-SR2 independent phenotype of the chimeric HIV virus carrying the MLV capsid and matrix proteins. We reanalyzed the HIV-1 N74D capsid mutant in cells transiently or stably depleted of transportin-SR2 and confirm that the N74D capsid mutant is independent of TRN-SR2 when pseudotyped with the vesicular stomatitis virus glycoprotein (VSV-G). Remarkably, although somewhat less dependent on TRN-SR2 than wild type virus, the N74D capsid mutant carrying the wild type HIV-1 envelope required TRN-SR2 for efficient replication. By pseudotyping with envelopes that mediate pH-independent viral uptake including HIV-1, measles virus and amphotropic MLV envelopes, we demonstrate that HIV-1 N74D capsid mutant viruses retain partial dependency on TRN-SR2. However, this dependency on TRN-SR2 is lost when the HIV N74D capsid mutant is pseudotyped with envelopes mediating pH-dependent endocytosis, such as the VSV-G and Ebola virus envelopes.

**Conclusion:**

Here we discover a link between the viral entry of HIV and its interaction with TRN-SR2. Our data confirm the importance of TRN-SR2 in HIV-1 replication and argue for careful interpretation of experiments performed with VSV-G pseudotyped viruses in studies on early steps of HIV replication including the role of capsid therein.

## Background

Retroviruses stably integrate the DNA copy of their RNA genome into the host cell chromatin. However, there are marked differences between the distinct families of retroviruses regarding their capacity to replicate in non-dividing cells. The *lentivirinae *such as the human immunodeficiency virus type 1 (HIV-1) can infect dividing and non-dividing cells such as macrophages, dendritic cells or CD4+ memory T-cells [1]. Rous sarcoma virus (RSV) can also infect non-dividing cells such as neurons or growth-arrested cells, but with less efficiency than HIV [2]. In contrast, the γ-retrovirus Moloney murine leukemia virus (MLV) infects only dividing cells efficiently [3]. To date, this difference cannot be explained. The prevailing hypothesis has been that lentiviruses adopt a specific mechanism for active nuclear import through the nucleopore, and that other retroviruses must depend on the breakdown of the nuclear membrane during mitosis for chromatin access in order to achieve integration [3-5]. More recently, a role for retroviral capsid was proposed in replication determination in non-dividing cells [6,7]. After HIV entry in the target cell, the viral core is released into the cytoplasm. On its way to the nucleus, viral capsid (CA) is shed from this nucleoprotein complex, containing both viral and cellular proteins, in an ill-defined process called uncoating (for a recent overview see [8]). Meanwhile the viral enzyme reverse transcriptase (RT) transcribes the RNA genome into a cDNA copy.

After reverse transcription, the preintegration complex (PIC) is transported through the nuclear pore complex (NPC). The NPC is a specialized channel ~40 nm in diameter [9] that supports passive diffusion of small molecules and ions and facilitates receptor-mediated translocation of proteins and ribonucleoprotein complexes above 40 kDa. Since the HIV-1 PIC is a nucleoprotein complex with an estimated diameter of 56 nm [10], it requires conformational changes and active transport through the NPC. Many attempts have been made to determine the viral and cellular factors mediating nuclear import of the HIV PIC (for a review see [11]). Viral protein R (Vpr), matrix protein (MA), integrase (IN) and the DNA flap have each been proposed as the main viral determinant for nuclear trafficking of the PIC, but these findings were not readily reproduced in subsequent studies. As cellular cofactors, importin-α/importin-β [12-15] and importin-7 [16-19] have been investigated as PIC transporters, but their role in HIV replication has not been thoroughly validated or confirmed. Also, importin-α3 has very recently been implicated in HIV nuclear import [20].

Recently, we identified the cellular protein transportin-SR2 (TRN-SR2, TNPO3, transportin 3), encoded by the *TNPO3 *gene, as the nuclear import factor of HIV [21]. Two genome-wide RNAi screens [22,23], but not others [24,25] also identified TRN-SR2 as a cofactor of HIV replication. Transportin-SR2 (TRN-SR2) was first identified as an important nuclear import factor for phosphorylated splicing factors of a family of serine/arginine-rich proteins (SR proteins) [26-28]. It has also been shown that TRN-SR2 imports other proteins not belonging to the SR protein family [29]. We identified TRN-SR2 as a binding partner of HIV-1 integrase in a yeast two-hybrid screen [21], and reverse yeast two-hybrid screening demonstrated that none of the other HIV proteins directly interacts with TRN-SR2. In cells transiently or stably depleted of TRN-SR2, HIV replication was severely hampered due to a defect in the nuclear import of the HIV PIC [21]. Using GFP-labeled HIV, a direct effect of TRN-SR2 on the nuclear import of PICs was also visualized. Finally, TRN-SR2 was required for HIV infection of both dividing and non-dividing cells, implying that a similar nuclear import pathway is used in different stages of the cell cycle.

A recent study confirmed the effect of TRN-SR2 knockdown on HIV-1 vector transduction [30]. In that study the specificity for different retroviral vectors and the direct interaction of TRN-SR2 with the integrase proteins from different retroviruses were examined, and the authors corroborated the direct interaction between recombinant TRN-SR2 and HIV-1 IN. Although TRN-SR2 was found to be a rather prolific IN binder, displaying affinity for multiple retroviral integrases, no clear correlation between the interactions of various integrases with TRN-SR2 and dependence on TRN-SR2 during viral vector transductions was observed. In addition, a chimeric reporter virus composed of both HIV and MLV proteins (MHIV) carrying the MLV MA, p12 and CA proteins instead of the HIV-1 MA and CA proteins [6,31], which was also pseudotyped with the vesicular stomatitis virus glycoprotein (VSV-G) envelope, appeared to be insensitive to TRN-SR2 knockdown. Although no evidence was provided that TRN-SR2 and CA physically interact, it was proposed that the TRN-SR2 dependency of HIV-1 infection is mediated by CA and not by HIV-1 integrase [30]. In a follow up study, the role of CA in the TRN-SR2 requirement of HIV-1 replication was examined in more detail [32]. Ectopic expression of a C-terminally truncated version of the cleavage and polyadenylation specific factor 6 (CPSF6) resulted in a block of HIV replication. An HIV-1 strain with a mutation in CA (N74D) was capable of escaping this phenotype. Interestingly, the VSV-G pseudotyped HIV-1 N74D CA mutant virus appeared to be independent of TRN-SR2 for infection of both dividing and non-dividing cells [32]. Here we enter the debate by re-examining whether HIV CA is involved in the TRN-SR2 requirement of HIV. We compared wild type and VSV-G pseudotyped viral vectors and studied the N74D CA mutant which was reported to be independent of TRN-SR2. To our surprise, the phenotype of the N74D CA mutant virus appeared to be dependent on the viral entry route. Whereas the mutant virus was insensitive to TRN-SR2 depletion when pseudotyped with VSV-G, the same mutant proved to be still dependent on TRN-SR2, although to a somewhat lesser extent, when retaining the HIV envelope. Our results are suggestive of a role for capsid mutations having an indirect effect on the interaction between HIV and TRN-SR2, probably by affecting the processes of uncoating or docking to the nuclear pore that precede the previously demonstrated interaction between IN and TRN-SR2.

## Results

### Lentiviral specificity of TRN-SR2

**Figure 1:**
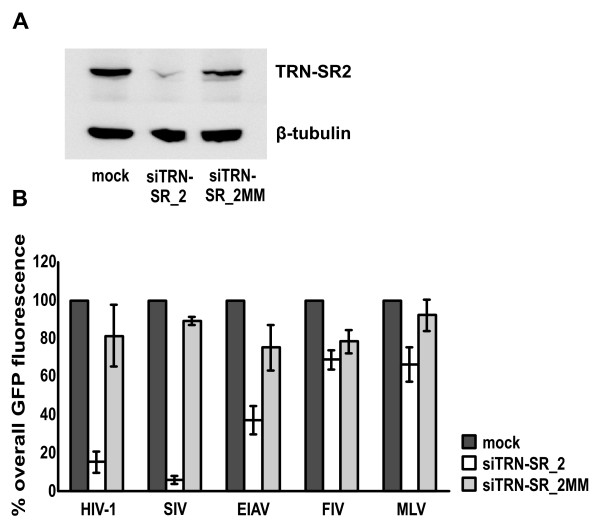
**Effect of TRN-SR2 knockdown on transduction efficiency of various retroviral vectors**. (A) TRN-SR2 knockdown in HeLaP4 cells 3 days post siRNA transfection visualized by western blotting. β-tubulin was detected as a control for equal loading. (B) HeLaP4 cells were transfected with siRNAs for knockdown of TRN-SR2 (siTRN-SR_2), with a mismatched control siRNA (siTRN-SR_2MM) or were mock transfected (mock). Three days post transfection cells were transduced with different VSV-G pseudotyped retroviral vectors encoding GFP. The overall GFP fluorescence was measured by flow cytometry and is expressed as the percentage relative to the control cell values. Results represent mean values ± standard deviation (SD) of at least 3 independent experiments each performed in triplicate. In each experiment newly produced viral vectors were used.

We previously identified TRN-SR2 as an important cellular cofactor mediating HIV-1 nuclear import [21]. HIV-2 was also dependent on TRN-SR2, although MLV did not appear to be dependent. Here we verified whether TRN-SR2 acts as a lentivirus-specific nuclear import factor. We transduced HeLaP4 cell lines transiently depleted of TRN-SR2 with different retroviral vectors (Figure 1), and a mismatch siRNA was run in parallel to exclude off-target effects while mock transfected cells were used as controls for the transfection procedure. TRN-SR2 knockdown was verified by western blot (Figure 1A). Three days after siRNA transfection cells were transduced with concentrated VSV-G pseudotyped viral vectors derived from HIV-1, SIV (simian immunodeficiency virus), EIAV (equine infectious anemia virus), FIV (feline immunodeficiency virus) or MLV. Vector preparations were adjusted to yield 30-60% GFP positivity in mock transfected cells. Three days after transduction cells were fixed and analyzed for the overall GFP fluorescence by flow cytometry (Figure 1B). After TRN-SR2 knockdown, transduction by either HIV-1 or SIV vectors was inhibited up to 90% and 95%, respectively. The EIAV vector was also sensitive to TRN-SR2 depletion, although to a lesser extent (50% inhibition of transduction efficiency). Transductions by the FIV and MLV vectors were modestly affected (12% and 29% inhibition, respectively) when compared to mismatch siRNA-transfected cells. From this analysis, we conclude that TRN-SR2 is a cellular cofactor important for transduction by some, but not all VSV-G pseudotyped lentiviral vectors.

The central DNA flap is a structure in the reverse transcribed DNA genome of lentiviruses that is absent from retroviruses like MLV [33,34]. Since the DNA flap has been implicated in HIV nuclear import [35-40], we examined whether the central DNA flap might be important for the TRN-SR2 requirement of HIV-1. HeLaP4 cell lines transiently depleted of TRN-SR2 and control cells were challenged with 3 dilutions of HIV-1-derived VSV-G pseudotyped lentivectors carrying a cPPT/CTS sequence in sense (WT) or antisense orientation (Flap-). In antisense orientation, the cPPT/CTS sequence does not yield a functional flap. Vectors lacking a functional flap are known to display a 3- to 6-fold reduction in transduction efficiency [37]. Three days after transduction, overall GFP fluorescence was measured by flow cytometry (Additional file 1: Figures S1A and S1B). The vector dilutions yielded 90%, 60% or 20% GFP positive control cells, respectively. Transduction by the lentiviral vectors with (Additional file 1: Figure S1A) or without DNA flap (Additional file 1: Figure S1B) was inhibited up to 70% in TRN-SR2 depleted cells and at all dilutions used. Next, we tested the effect of DNA flap mutations on the multiple-round infectivity of HIV-1 virus strain NL4-3 in TRN-SR2 depleted HeLaP4 cells (Additional file 1: Figures S1C and S1D). We infected HeLaP4 cells transiently depleted of TRN-SR2 and control cells with 3 dilutions of infectious HIV-1_NL4-3_LAI cPPT wild type virus (WT) (Additional file 1: Figure S1C) and HIV-1_NL4-3_LAI cPPTD (Flap-) virus (Additional file 1: Figure S1D). The latter contains a mutated cPPT sequence which prevents formation of the DNA flap during reverse transcription and shows a 10- to 100-fold replication defect depending on the viral infection dose [35,38]. The HeLaP4 cells contain a β-galactosidase (β-gal) reporter gene under control of the HIV-1 LTR promoter. Three days after infection β-galactosidase activity was measured. The data demonstrate reduced infectivity of the flap-negative virus, which becomes more apparent at lower MOIs as was previously reported [35,38]. TRN-SR2 knockdown inhibited replication of wild type and flap-negative virus to the same extent (up to 80% inhibition) demonstrating that the DNA flap is not required for the TRN-SR2 dependency of HIV-1 replication.

### The MLV capsid confers TRN-SR2 independence to chimeric HIV-1

Next we investigated which viral proteins, present in the PIC, are responsible for the TRN-SR2 independent phenotype displayed by the MLV vector. We used the HIV-MLV chimeric viruses (MHIV) previously constructed by the Emerman group [6,31]. In MHIV-mMA12CA the HIV MA and CA proteins are replaced by the MLV MA, CA and p12 proteins. In MHIV-mIN, the HIV IN protein is replaced by the MLV IN. MHIV-mMA12CA cannot infect non-dividing cells, but MHIV-mIN infects non-dividing cells as well as dividing cells [6,31]. Since these viruses are poorly infectious, VSV-G pseudotyping of the chimeric viruses during productions is absolutely required to obtain infectious virions. Construction of MLV-based chimeric proviruses did not generate infectious virions [6].

**Figure 2:**
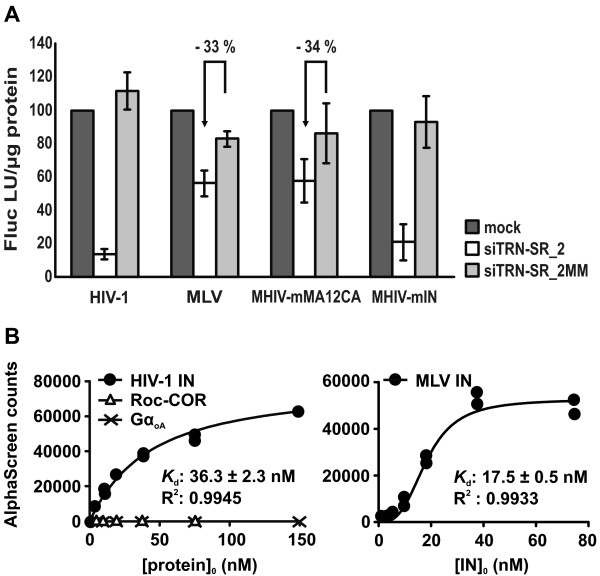
**HIV containing MLV capsid is largely TRN-SR2 independent, HIV with MLV integrase is not**. (A) HeLaP4 cells depleted of TRN-SR2 (siTRN-SR_2) and control cells (mock and siTRN-SR_2MM) were infected with VSV-G pseudotyped HIV-1 single-round virus, MLV vector, or with the chimeric viruses MHIV-mMA12CA or MHIV-mIN. In MHIV-mMA12CA the HIV MA and CA proteins are replaced by the MLV MA, CA and p12 proteins. In MHIV-mIN the HIV IN protein is replaced by MLV IN. Three days post infection cells were lysed and Fluc activity was measured and normalized to the total amount of protein in the cell lysates. Results are shown as the mean values of relative light units per μg protein (Fluc RLU/μg protein) ± SD compared to mock transfected cells and represent 2 independent experiments each performed in triplicate. The arrows indicate the relative inhibition of infectivity in TRN-SR2 depleted cells compared to mismatch siRNA tranfected cells. (B) Direct interactions between recombinant GST-TRN-SR2 and His_6_-tagged HIV-1 IN or MLV IN were measured by AlphaScreen. As negative controls for binding to GST-TRN-SR2 both His_6_-Gα_oA _and His_6_-Roc-COR were used. 10 nM of GST-TRN-SR2 was incubated with different concentrations of His_6_-tagged proteins and complexes were bound to glutathione donor beads and nickel-chelate acceptor beads. Light emission was measured using an EnVision Multilabel Reader. The apparent equilibrium dissociation constants (*K*_d_) were calculated with GraphPad Prism 5 and are indicated on the graphs.

We evaluated the effect of siRNA-mediated TRN-SR2 knockdown in HeLaP4 cells on infection by both MHIV chimeric viruses (Figure 2A). We used VSV-G pseudotyped single-round MHIV-mMA12CA and MHIV-mIN viruses and their parental HIV-1 and MLV vector, all expressing the firefly luciferase reporter gene (Fluc). HeLaP4 cells transiently depleted of TRN-SR2 and control cells were infected with concentrated VSV-G pseudotyped viral stocks. Luciferase activities were measured 3 days post infection and were normalized to the levels in mock transfected control cells (Figure 2A). As previously reported [21-23,30], the MLV vector displayed only modest sensitivity to TRN-SR2 knockdown (33% inhibition of infectivity in TRN-SR2 knockdown cells compared to mismatch siRNA transfected cells). Surprisingly, the MHIV-mMA12CA chimeric virus was only partially sensitive to TRN-SR2 knockdown (34% inhibition in TRN-SR2 knockdown cells compared to mismatch siRNA transfected cells). In contrast, both the MHIV-mIN virus and the parental HIV-1 reporter virus were severely impaired by TRN-SR2 knockdown (77% and 87% inhibition, respectively). These results are comparable to those described by Krishnan and colleagues [30]. Swapping of HIV MA and CA proteins with those of MLV apparently interferes with the requirement for TRN-SR2 during infection but replacing the IN of HIV-1 by that of MLV does not alter the TRN-SR2 dependency of the chimeric virus.

Two alternative explanations for these results are possible. The viral CA may determine the interaction between TRN-SR2 and the HIV-1 PIC as was proposed by Krishnan *et al*. [30]; and by replacing the HIV-1 CA and MA proteins by their non-interacting MLV counterparts, this interaction could be inhibited, rendering infection of the MHIV-mMA12CA virus partially independent of TRN-SR2. Substituting the integrases in this case would have no effect. Alternatively, TRN-SR2 can interact with both HIV-1 and MLV IN and, as a result, the MHIV-mIN virus would remain dependent on TRN-SR2. However, recombinant His-tagged HIV-1 IN could pull down endogenous TRN-SR2 in cellular lysates, but recombinant His-tagged MLV IN could not [21]. Still, this interaction could have gone undetected due to low concentrations of endogenous TRN-SR2 in the cell lysate which are difficult to detect by western blot alone. Therefore, we reinvestigated the direct protein-protein interaction using AlphaScreen technology and recombinant GST-TRN-SR2 and IN-His_6 _(Figure 2B). As negative controls for binding to GST-TRN-SR2, we used two different His_6_-tagged proteins; His_6_-Gα_oA_, the His_6_-tagged human heterotrimeric G protein α oA subunit [41], and His_6_-Roc-COR, a His_6_-tagged GTPase Ras of complex proteins (Roc) domain in tandem with its C-terminal domain of Roc (COR) of the leucine rich repeat kinase 2 protein (LRRK2) from *Chlorobium tepidum *[42]. The different His_6_-tagged proteins were titrated against a fixed concentration of GST-TRN-SR2 (10 nM). As expected, no interaction between His_6_-Gα_oA _or His_6_-Roc-COR and GST-TRN-SR2 was detected under our assay conditions (Figure 2B). In this assay, we did observe binding of GST-TRN-SR2 to both His_6_-tagged HIV-1 IN and MLV IN with an apparent *K*_d _of 36.3 ± 2.3 nM for HIV-1 IN and an even lower *K*_d _of 17.5 ± 0.5 nM for MLV IN, an interaction also observed by Krishnan *et al*. [30]. Our data are consistent with the hypothesis that TRN-SR2 binds to both HIV-1 and MLV IN in the context of a viral PIC, explaining the TRN-SR2 dependency of MHIV-mIN, the chimeric HIV virus containing MLV IN. However, this does not explain the TRN-SR2 independent phenotype of the MLV vector and the MHIV-mMA12CA virus. Moreover, a CA mutant virus (HIV-1 N74D) has recently been described to be insensitive to TRN-SR2 knockdown [32]. These findings prompted us to investigate in more detail a possible role of HIV-1 CA in the TRN-SR2 requirement of HIV-1 replication.

### The HIV-1 N74D CA mutant virus still requires TRN-SR2 for efficient infection

**Figure 3:**
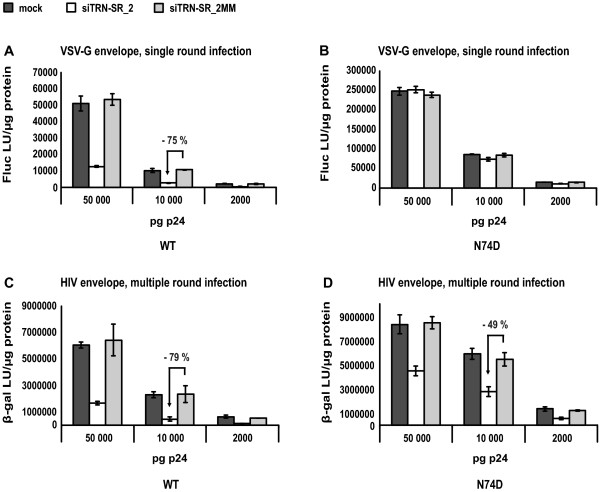
**The HIV-1 N74D CA mutant remains partially dependent on TRN-SR2 when carrying the HIV envelope**. (A) HeLaP4 cells depleted of TRN-SR2 (siTRN-SR_2) and control cells (mock and siTRN-SR_2MM) were challenged using 3 dilutions of VSV-G pseudotyped HIV-1 NL4-3 (WT) or (B) HIV-1 NL4-3 N74D CA mutant (N74D) luciferase reporter viruses. Three days post infection Fluc activity was measured and normalized to the total amount of protein in the cell lysates. Graphs show the mean values of Fluc light units per μg protein (Fluc LU/μg protein) ± SD of one representative experiment out of two performed in triplicate. (C) Same as in (A), but multiple-round viruses HIV-1 NL4-3 (WT) or (D) HIV-1 NL4-3 N74D CA mutant (N74D) carrying the HIV-1 envelope were used for infections. Infectivity was measured by β-gal activity 72 hours post infection. The arrows indicate the relative inhibition of infectivity in TRN-SR2 depleted cells compared to mismatch siRNA tranfected cells.

A VSV-G pseudotyped HIV-1 N74D CA mutant virus was recently reported to be insensitive to TRN-SR2 knockdown [32]. Krishnan *et al*. [30] hypothesized that the TRN-SR2 dependency of HIV-1 is dictated by HIV-1 CA instead of IN. To test this hypothesis, we infected HeLaP4 cells transiently depleted of TRN-SR2 with VSV-G pseudotyped wild type and N74D CA mutant luciferase reporter viruses (Figures 3A and 3B) or with replication competent HIV-1 NL4-3 wild type and N74D mutant virus (Figures 3C and 3D). The infectivity of the VSV-G pseudotyped wild type and N74D CA mutant luciferase reporter viruses was measured by Fluc activity. Interestingly, after normalization of the virus stocks based on p24 measurements (PerkinElmer, HIV-1 p24 ELISA kit), the VSV-G pseudotyped N74D CA mutant virus appeared 5-fold more infectious (compare Figures 3A and 3B). In repeated infection experiments, the VSV-G pseudotyped N74D mutant virus consistently displayed 5- to 10-fold higher luciferase counts than pseudotyped wild type virus (data not shown). We confirmed the TRN-SR2 independent phenotype of the VSV-G pseudotyped N74D CA mutant (Figure 3B) in comparison to the pseudotyped wild type virus (75% reduction of viral infectivity on average in the TRN-SR2 knockdown cells compared to the mismatch siRNA transfected cells) (Figure 3A). Similar results were obtained with a β-galactosidase readout (data not shown).

Subsequently, we infected HeLaP4 cells transiently depleted of TRN-SR2 with replication competent wild type and N74D mutant viruses. Both viruses carried the HIV-1 envelope proteins gp120 and gp41. To allow multiple round replication, three days after infection β-galactosidase activity was measured as readout for viral infectivity. Virus stocks were normalized for p24 content (PerkinElmer p24 ELISA kit). The N74D mutant again yielded higher β-galactosidase counts (compare Figures 3C and 3D), although the difference in infectivity was less pronounced (1.5-fold higher infectivity than wild type virus) than with the VSV-G pseudotyped N74D CA mutant (5- to 10-fold higher infectivity than wild type virus, compare Figures 3A and 3B). In repeated experiments using viruses carrying the HIV-1 envelope, we consistently observed a 1.5- to 3-fold higher infectivity of the N74D CA mutant measured via β-galactosidase activity (data not shown). We observed that both HIV-1 NL4-3 WT virus (Figure 3C) and the N74D CA mutant (Figure 3D) required TRN-SR2 for efficient infection of HeLaP4 cells (average reduction of infectivity of 80% and 50% in TRN-SR2 knockdown cells, respectively), although the N74D CA mutant virus was less sensitive to TRN-SR2 depletion. Nevertheless, a prominent alteration in the TRN-SR2 dependency of the N74D CA mutant was observed when using the VSV-G envelope (complete insensitivity to TRN-SR2 knockdown, Figure 3B) or the HIV-1 envelope (intermediate sensitivity to TRN-SR2 knockdown, Figure 3D). As these findings suggest, the difference in TRN-SR2 dependency displayed by the N74D CA mutant in the multiple round compared with the single round infections was dependent on the envelope proteins; we investigated prolonged multiple round replication of the HIV-1 N74D CA mutant carrying a wild type envelope using HeLaP4 cells stably depleted of TRN-SR2 knockdown.

**Figure 4:**
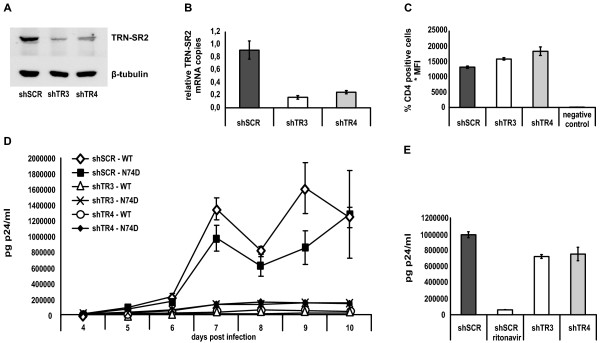
**Stable TRN-SR2 depletion inhibits multiple round infection by HIV-1 WT and N74D CA mutant virus**. (A) Western blot showing TRN-SR2 levels in HeLaP4 cells stably depleted of TRN-SR2 by shRNA expressing vectors (shTR3 and shTR4) and control cells expressing a scrambled (shSCR) shRNA. β-tubulin was detected as loading control. (B) TRN-SR2 knockdown in the shTR3 and shTR4 HeLaP4 cells measured by QPCR. TRN-SR2 mRNA levels are normalized for β-actin. Shown are mean values ± SD of triplicate measurements. (C) Analysis of CD4 expression levels of the shSCR, shTR3 and shTR4 HeLaP4 cells by anti-CD4 immunostaining and flow cytometry. 293T cells were analyzed in parallel as control for non-specific staining. Averages of triplicate samples ± SD are shown. (D) Stably TRN-SR2 depleted cells and control cells were infected with 6 × 10^4 ^pg p24 of infectious HIV NL4-3 (WT) or HIV-1 NL4-3 N74D CA mutant virus (N74D). From four days post infection on supernatants were sampled daily for p24 measurements. One of three independent experiments each performed in duplicate is shown. (E) shTR3, shTR4 and shSCR cells were transfected with 1 μg of pNL4-3 molecular clone plasmid. 24 hours post transfection supernatants were analyzed for p24 production. 5 μM of ritonavir was used as a positive control of inhibition of p24 production. Shown are mean values ± SD of one experiment out of two performed in triplicate.

We generated stable TRN-SR2 knockdown cell lines by transducing HeLaP4 cells with one of two different lentiviral vectors expressing shRNA targeting the TRN-SR2 mRNA (shTR3 and shTR4) and a control cell line using a control vector expressing a scrambled shRNA (shSCR). TRN-SR2 knockdown was verified with Western blotting (Figure 4A) and QPCR (Figure 4B). When visualized by Western blotting, TRN-SR2 knockdown in the shTR3 or shTR4 HeLaP4 cells was comparable to the level of knockdown obtained by transient transfection of siTRN-SR_2 (compare Figures 4A and 1A). When measured by QPCR, expression of shTR3 or shTR4 decreased the amount of TRN-SR2 mRNA copies for 80% or 70% respectively in comparison with control cells (Figure 4B). Using immunostaining and FACS analysis of the CD4 surface receptor expressed by the TRN-SR2 depleted cells and control cells, comparable CD4 expression levels in the knockdown cells and control cells were observed (Figure 4C). As expected, no CD4 expression was observed in 293T cells which were used as a negative control to exclude non-specific binding of the anti-CD4 antibody. The stable TRN-SR2 knockdown and control cell lines were challenged with wild type HIV-1 NL4-3 and N74D CA mutant virus in a multiple-round infection (Figure 4D), with the inocula normalized for p24 content. Both viruses replicated with similar kinetics in the shSCR control cells. The replication of both the HIV-1 wild type virus and the HIV-1 N74D CA mutant was severely impaired up to 10 days post infection in both shTR3 and shTR4 HeLaP4 cell lines stably depleted of TRN-SR2, although the N74D CA mutant was somewhat less sensitive to TRN-SR2 knockdown (10-fold inhibition in the shTR3 HeLaP4 cells compared to shSCR cells) than the wild type virus (70-fold inhibition in the shTR3 expressing cells compared to shSCR cells). To exclude a non-specific effect of TRN-SR2 knockdown or the expression of the different shRNAs on the late steps of viral replication, we transfected the shSCR, shTR3 and shTR4 HeLaP4 cell lines with the viral molecular clone pNL4-3 and measured the p24 levels in the supernatant 24 hours after transfection (Figure 4E). Although a slight inhibition of the p24 production was observed in the TRN-SR2 knockdown cell lines, this difference could not account for the potent inhibition of HIV replication in the TRN-SR2 depleted cells. These results confirm our previous finding that TRN-SR2 depletion does not inhibit the late steps of HIV replication [21] and exclude non-specific effects on the late steps of HIV replication in the shTR3 and shTR4 expressing HeLaP4 cells compared to the shSCR control cell line. Together, these results show that the HIV-1 N74D CA mutant virus still requires TRN-SR2 for efficient infection in HeLaP4 cells.

**Figure 5:**
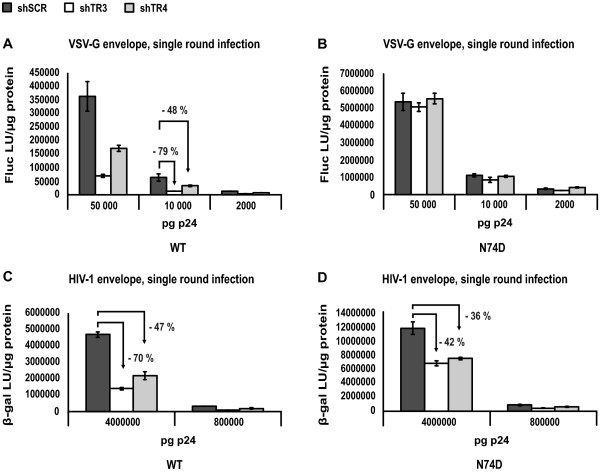
**Stable TRN-SR2 depletion inhibits single round infection by HIV-1 WT and N74D CA mutant virus**. (A) shTR3, shTR4 and shSCR HeLaP4 cells were challenged using 3 dilutions of VSV-G pseudotyped HIV-1 NL4-3 (WT) or (B) HIV-1 NL4-3 N74D CA mutant (N74D) luciferase reporter viruses. Two days post infection Fluc activity was measured and normalized to total protein amounts. Graphs show the mean values of Fluc light units per μg protein (Fluc LU/μg protein) ± SD of one representative experiment out of two performed in triplicate. (C) Same as in (A), but 2 dilutions of multiple-round viruses HIV-1 NL4-3 (WT) or (D) HIV-1 NL4-3 N74D CA mutant (N74D) carrying the HIV-1 envelope were used in the presence of 5 μM ritonavir to ensure single round infection. Infectivity was measured by β-gal activity 72 hours post infection. The arrows indicate the relative inhibition of infectivity in the shTR3 and shTR4 HeLaP4 cells compared to shSCR control cells.

Next, we wondered whether the inhibition of the N74D CA mutant by stable TRN-SR2 knockdown in the multiple round infection experiments could be mirrored in single round infection assays. We tested the infectivity of VSVG-pseudotyped wild type and N74D CA mutant virus, or replication competent wild type and N74D CA mutant viruses in single-round infection experiments in the HeLaP4 cells stably depleted of TRN-SR2 and in control cells. For these experiments, we normalized the virus stocks for p24 content and for RT activity (see the materials and methods section, paragraph infection and transduction). Various amounts of VSV-G pseudotyped HIV-1 NL4-3 and HIV-1 NL4-3 N74D CA mutant luciferase reporter viruses were used to infect the shSCR-, shTR3- and shTR4-expressing HeLaP4 cells (Figures 5A and 5B). Infectivity was measured by Fluc activity. In these experiments, the pseudotyped N74D CA mutant virus again proved to be more infectious than the wild type reporter virus (typically 10- to 15-fold). The results were comparable to the experiments using HeLaP4 cells transiently depleted of TRN-SR2 (Figure 3), excluding possible non-specific effects on viral infectivity in the stable TRN-SR2 knockdown cells due to off-target effects or selection. The VSV-G pseudotyped HIV-1 N74D CA mutant (Figure 5B), in contrast to the VSV-G pseudotyped wild type virus (Figure 5A), did not require TRN-SR2 for infection as was described in [32]. Single round infectivity of the VSV-G pseudotyped wild type virus was less inhibited in the shTR4 TRN-SR2 knockdown cells (50% inhibition on average) compared to the shTR3 expressing TRN-SR2 knockdown cells (80% inhibition on average). This difference is likely due to the difference in the extent of TRN-SR2 knockdown in these different cell lines as measured by QPCR (compare Figures 5A and 4B). Next, we infected the HeLaP4 cells stably depleted of TRN-SR2 and control cells with two different dilutions of replication competent HIV-1 NL4-3 and N74D CA mutant virus normalized for p24 values (and RT activity) (Figures 5C and 5D). Single round infections were performed in the presence of 5 μM of the protease inhibitor ritonavir. β-galactosidase activity was measured as readout for viral infectivity. The N74D CA mutant was 2.5-fold more infectious than wild type HIV-1 in these experiments, corresponding to the increase in infectivity we observed in the experiments using transient siRNA-mediated knockdown of TRN-SR2 (Figures 3C and 3D). In the shTR3 HeLaP4 cells, we observed an average reduction of 70% of wild type HIV-1 virus infection compared to shSCR cells, and in the shTR4 HeLaP4 cells an average reduction of 50% (Figure 5C), comparable to the experiments using VSV-G pseudotyped wild type virus (Figure 5A). Infection by the N74D CA mutant was inhibited 40% on average in the shTR3 cells, and 35% on average in the shTR4 cells (Figure 5D). These results point to a partial TRN-SR2 dependency of the N74D CA mutant virus when carrying the HIV-1 envelope.

### Different entry routes influence TRN-SR2 dependency of the HIV-1 N74D CA mutant virus

The major endocytic pathways include pH-dependent clathrin-mediated endocytosis, pH-independent caveolae-mediated endocytosis, clathrin- and caveolae-independent endocytosis, macropinocytosis and phagocytosis (for a review see [43]). To investigate whether endocytosis in general renders the HIV-1 N74D CA mutant insensitive to TRN-SR2 knockdown, we produced wild type and N74D CA mutant NL4-3 luciferase reporter virus pseudotyped with the HIV-1 envelope glycoproteins, VSV-G, or viral envelopes derived from the measles virus, amphotropic MLV or Ebola virus. Both the HIV-1 envelope and the measles virus envelope mediate viral entry via pH-independent fusion of the viral and cellular membranes [44], although HIV-1 virions are also proposed to enter cells via pH-independent endocytosis leading to unproductive infection [45-47], or via endocytosis and subsequent dynamin-dependent fusion with endosomes [48]. The modes of entry of amphotropic MLV (MLVampho) and the highly pathogenic Ebola virus have been the subject of debate, but recent studies showed that MLVampho enters the cell via a pH-independent, caveola-dependent endocytic pathway [49] while the Ebola virus enters through pH-dependent clathrin-mediated endocytosis [50]. VSV-G pseudotyped viral particles enter target cells via pH-dependent endocytosis, although the role of clathrin in this process is not well understood [50-52]. Although we observed previously (Figure 4D) that the TRN-SR2 dependent phenotype of HIV-1 is more pronounced in prolonged multiple round infections, pseudotyping of the wild type and N74D CA mutant reporter viruses with different viral envelopes obliged single round infection experiments.

**Figure 6:**
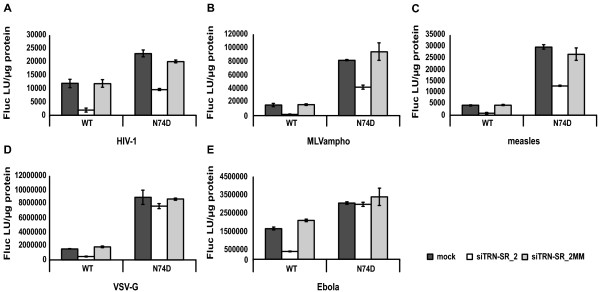
**The viral entry route influences the TRN-SR2 dependency of the HIV-1 N74D CA mutant**. HeLaP4 cells depleted of TRN-SR2 (siTRN-SR_2) and control cells (mock and siTRN-SR_2MM) were challenged using 3 dilutions of pseudotyped HIV-1 NL4-3 (WT) or HIV-1 NL4-3 N74D CA mutant (N74D) luciferase reporter viruses. Viruses were pseudotyped with various envelopes derived from HIV-1 (A), amphotropic MLV (B), measles virus (C), VSV (D) or Ebola virus (E). Three days post infection Fluc activity was measured and normalized to the total amount of protein in the cell lysates. Graphs show the mean values of triplicate measurements of Fluc light units per μg protein (Fluc LU/μg protein) ± SD of a representative experiment.

HeLaP4 cells transiently depleted of TRN-SR2 and control cells were challenged with the differently pseudotyped WT and N74D CA mutant reporter viruses and infectivity was measured using the Fluc reporter protein activity as readout. The infectivities of the pseudotyped viruses were in a similar range in control cells when envelopes mediating the same entry route were used (Figure 6). When the HIV-1 N74D CA mutant reporter virus was pseudotyped with VSV-G (Figure 6D) or the Ebola envelope (Figure 6E), infections were not impaired by TRN-SR2 knockdown in contrast to wild type reporter virus. However, when the viral particles were pseudotyped with the HIV-1 envelope (Figure 6A), the MLVampho envelope (Figure 6B) or the measles virus envelope (Figure 6C), the N74D CA mutant was still dependent on TRN-SR2 (50% inhibition of infection in TRN-SR2 depleted cells), although not as dependent as the wild type virus (80-90% inhibition of infection). These findings show that the HIV-1 N74D CA mutant virus relies much less on TRN-SR2 when entering the target cells via pH-dependent endocytosis (VSV-G and Ebola envelope). After membrane fusion (HIV-1 and measles virus envelope) or pH-independent endocytosis (MLVampho envelope), the N74D CA mutant still requires TRN-SR2 for infection of HeLaP4 cells.

## Discussion

### Lentiviral specificity of TRN-SR2

We initially identified TRN-SR2 in a yeast two-hybrid screen searching for cellular binding partners of HIV-1 IN, and in the reverse screen no interaction with capsid was detected. Subsequently we showed that TRN-SR2 mediates nuclear import of the HIV-1 PIC [21]. Since one of the key features of lentiviruses is their ability to infect non-dividing cells, we questioned whether TRN-SR2 is a lentiviral-specific cofactor of HIV-1 replication. We challenged HeLaP4 cells depleted of TRN-SR2 with VSV-G pseudotyped retroviral vectors derived from HIV-1, SIV, EIAV, FIV or MLV, and the results obtained were comparable with recently reported data [30]. We also observed a TRN-SR2 independent phenotype for the FIV vector, and the MLV vector was only slightly sensitive to TRN-SR2 depletion (Figure 1). TRN-SR2 independent transduction by FIV, MLV and RSV derived vectors has been shown before [21-23,30]. Lee *et al*. reported that a pseudotyped FIV vector was insensitive to TRN-SR2 knockdown as well [32]. Together these data suggest that TRN-SR2 acts as a lentivirus-specific cofactor, although not all lentiviruses utilize it to the same extent. Because of our finding that VSV-G pseudotyping masks the TRN-SR2 requirement of the HIV-1 N74D CA mutant virus, it would be prudent to extend this study in a follow up project by using native viral envelopes or at least viral envelopes mimicking the natural entry pathway of each specific virus under study. HIV-1 and SIV are related lentiviruses and both enter target cells predominantly via membrane fusion. VSV-G pseudotyping did not alter the requirement of wild type HIV-1 for TRN-SR2 [21], although a single mutation in the CA protein (N74D) abolished the TRN-SR2 dependency of VSV-G pseudotyped HIV-1. Although VSV-G pseudotyped SIV was highly sensitive to TRN-SR2 knockdown, it would be interesting to test SIV with its viral envelope which mediates fusion-based entry. The EIAV virus is believed to enter the cell through pH-dependent, clathrin-mediated endocytosis [53], implying that VSV-G pseudotyping may actually resemble the natural entry pathway for this virus; the sensitivity for TRN-SR2 depletion of the pseudotyped EIAV vector may well reflect its natural dependency on TRN-SR2. The fusion-based mechanism of entry used by the FIV virus closely resembles that of HIV and SIV [54]. Both ecotropic and amphotropic MLV enter the cell through a pH-independent endocytic pathway [49,55]. When pseudotyping MLV and FIV one should take the specific entry pathways into account. At this stage, we cannot entirely exclude that FIV and MLV require TRN-SR2 for their replication since all experiments were performed with VSV-G pseudotyped vectors (our data and [30,32]). Krishnan and colleagues tried to correlate the binding affinities of recombinant TRN-SR2 for different retroviral integrases with the dependency on TRN-SR2 displayed by the corresponding viral vectors [30]. In the absence of any correlation, the authors concluded that IN must not play a dominant role in the TRN-SR2 requirement of HIV-1. However, our findings put into question the TRN-SR2-related phenotypes observed for the different viral vectors since all experiments described so far were performed using VSV-G pseudotyped viral particles and not the natural virus envelopes. Therefore, the conclusion that the requirement for TRN-SR2 during infection does not map to HIV-1 IN [30] may be premature, and requires further study on the retroviral specificity of TRN-SR2-mediated nuclear import using replicating viruses instead of vectors and the use of the respective host cells. Alternatively, one could pseudotype viral vectors while mimicking the natural entry mechanism to determine the role of TRN-SR2 in the replication of each particular virus. Although we don't provide direct evidence for an interaction between TRN-SR2 and HIV-1 IN in the context of a viral PIC during infection, we believe our results do not refute the hypothesis that IN plays a direct role in the TRN-SR2-mediated nuclear import of HIV.

Since the DNA flap has been implicated in nuclear import [35-40], we compared the infectivity of both HIV-1-derived VSV-G pseudotyped vectors and infectious viruses with or without a functional DNA flap in HeLaP4 cells depleted of TRN-SR2 (Additional file 1). The flap-negative vectors and viruses were as sensitive for TRN-SR2 depletion as the vectors and viruses harboring a functional DNA flap, ruling out an important role for the DNA flap in the TRN-SR2 requirement of HIV-1 replication.

### The MLV capsid renders pseudotyped HIV-1 largely independent of TRN-SR2

To understand the TRN-SR2 independent phenotype of the pseudotyped MLV vector observed in our previous experiments, we used pseudotyped MHIV chimeric viruses [6,31] to analyze the role of different viral factors in the TRN-SR2 dependency of HIV-1 (Figure 2). Both the pseudotyped MLV vector and the MHIV-mMA12CA chimera carrying the MLV CA and MA proteins in place of the corresponding HIV proteins were much less dependent on TRN-SR2 than the HIV-1 parental vector. Contrarily, the pseudotyped MHIV-mIN chimeric virus still required TRN-SR2 for infection of HeLaP4 cells. Similar results were recently reported [30].

There are two possible explanations for our observations: TRN-SR2 may bind to the HIV-1 CA and/or MA proteins but not to MLV CA and/or MA, or TRN-SR2 may bind to both HIV-1 IN and MLV IN. To investigate the latter hypothesis, we measured the direct protein-protein interaction between GST-TRN-SR2 and His-tagged HIV-1 IN or MLV IN. Both integrases strongly interact with TRN-SR2 and display similar dissociation constants (HIV-1 IN *K*_d_: 36.3 nM, MLV IN *K*_d_: 17.5 nM). While no physical interaction of TRN-SR2 and CA has yet been demonstrated, our data are consistent with TRN-SR2 interacting with the integrase protein during viral replication in the cell. We have also previously ruled out binding by TRN-SR2 to any other viral protein apart from HIV-1 IN by reverse yeast two-hybrid screening [21]. Although this result can explain the TRN-SR2 dependency of MHIV-mIN, it does not explain the TRN-SR2 independent phenotype of the MHIV-mMA12CA virus and the MLV vector. It is well known that the uncoating steps of HIV and MLV are quite different [56]. During the early steps of retroviral infection most of the CA proteins dissociate from the HIV nucleoprotein complexes of incoming virions, whereas a large amount of CA remains bound to the MLV nucleoprotein complexes [57,58]. The uncoating process may be the rate-limiting step determining further downstream events such as the interaction with TRN-SR2 and through that, the nuclear entry of the PIC. According to this hypothesis, the MLV capsid may mask or shield integrase from interaction with TRN-SR2. Alternatively, the presence of this capsid may alter docking of the PIC to the appropriate nucleoporin required for TRN-SR2 interaction, and, in so doing, the capsid may determine the vehicle selected to cross the nucleopore tunnel. Pseudotyping with VSV-G is required in studies with chimeric MHIV viruses to overcome their poor infectivity and to reach measurable titers [6,31]. As we have determined that pseudotyping of viruses may mask the role of TRN-SR2 during viral infection, we cannot exclude the possibility that MHIV-mMA12CA still requires TRN-SR2 for infection after viral entry mediated by fusion instead of endocytosis. Therefore, we cannot conclude, based on our experiments using the chimeric MHIV viruses and AlphaScreen protein-protein interaction assays, that the requirement for TRN-SR2 during infection does not map to HIV-1 IN, as was proposed by Krishnan *et al*., based on their comparable results [30].

### The viral entry route influences TRN-SR2 dependency of the HIV-1 N74D CA mutant

In an elegant study, Lee and colleagues describe how an HIV-1 CA mutant, N74D, renders HIV-1 resistant to a block in replication imposed by the ectopic expression of a C-terminally truncated version of CPSF6 [32]. Surprisingly, VSV-G pseudotyped HIV-1 N74D CA mutant virus was shown to efficiently infect both dividing and non-dividing cells depleted of TRN-SR2. In the same study, the nucleoporins (Nups) selected by pseudotyped HIV-1 were shown to also depend on the single residue change in CA. While pseudotyped wild type HIV-1 virus was inhibited by knockdown of Nup153 and Nup160, the pseudotyped HIV-1 N74D CA mutant virus was impaired to a greater extent by knockdown of Nup85, Nup155 and Nup160.

We have now demonstrated that the TRN-SR2 requirement for replication of the HIV-1 N74D CA mutant strictly depends on the viral envelope chosen (Figures 3, 4, 5 and 6). In a multiple round infection experiment using HeLaP4 cells stably depleted of TRN-SR2, which more closely resembles a natural occurring HIV infection than single round infection assays, the N74D CA mutant virus proved to be still dependent on TRN-SR2 (Figure 4D), although to a somewhat lesser extent than wild type HIV-1. When we pseudotyped viral particles with the VSV-G (Figures 3A, B, 5A, B and 6D) or the Ebola virus envelope (Figure 6E), which both mediate entry through pH-dependent endocytosis, the mutant virus proved completely independent of TRN-SR2. However, after pseudotyping the N74D CA mutant with envelopes derived from MLVampho (Figure 6B) or measles virus (Figure 6C) or in the presence of the wild type HIV-1 envelope (Figures 3C, D, 5C, D and 6A), the N74D CA mutant was intermediately impaired by TRN-SR2 knockdown. HIV-1 and measles virus enter cells by membrane fusion, while MLVampho uses a pH-independent, caveola-dependent endocytic route. Both the multiple round infections using p24 as readout (Figure 4D) and the infection experiments using bioluminescent reporter proteins to measure infectivity (Figures 3 and 5) suggest that the TRN-SR2 independent phenotype displayed by the VSV-G pseudotyped HIV-1 N74D CA mutant depends on the viral envelope mediating entry into the target cell and the trafficking route, rather than on a reduced interaction between CA and TRN-SR2 as was hypothesized by Krishnan *et al*. [30]. Intriguingly, the partial dependence on TRN-SR2 suggests that the N74D CA mutant is able to use an alternative nuclear import pathway besides TRN-SR2-mediated nuclear trafficking, especially when entering the cell via pH-dependent endocytosis.

The reason why certain CA mutations abolish the requirement for TRN-SR2 of HIV-1, especially when pseudotyped, is currently unclear. It is known that the HIV-1 CA protein interacts with different cellular cofactors like cyclophilin A and restriction factors like TRIM5α [59]. It is plausible that the N74D CA mutation could inhibit or stimulate certain interactions of the incoming viral cores with cellular cofactors or restriction factors, thereby altering the structure of the PIC and shielding the interaction with TRN-SR2. This may also explain the higher infectivity of the HIV-1 N74D CA mutant virus compared to wild type virus (Figures 3, 4, 5 and 6). Overall, the N74D CA mutant is more infectious than the wild type virus in our hands, especially when pseudotyped with the VSV-G envelope, and this higher infectivity seems more pronounced in single round infection assays using bioluminescence to measure infectivity.

It is also known that endocytosis-based uptake of virions in target cells, mediated by the VSV-G envelope, changes the trafficking route followed by the incoming viral particles during early steps of infection. After entering the cell, viruses need to penetrate cortical actin, the dense layer of actin microfilaments underneath the plasma membrane [60]. Endocytosis naturally by-passes the cortical actin barrier and transports the maturing viral cores deeper into the cytoplasm before they are released from the endocytic endosomes [60,61]. It is possible that this different way of entry guides the incoming viral cores to other trafficking routes along the microtubuli. These re-routed PICs may dock eventually to different nucleoporins and interact with other importins regulating nuclear import. Anderson and Hope also suggest that the HIV-1 CA may direct viral nucleoprotein complexes to a cytoplasmic pathway leading to efficient NPC trafficking before being shed from the incoming viral nucleoprotein complex [61]. Hypothetically, the N74D mutant CA could direct PICs to an alternative transport pathway and a different compartment of the nuclear membrane where an unknown alternative import pathway is used. This may also explain the reduced TRN-SR2 dependency of the N74D CA mutant when carrying the HIV-1 envelope.

The velocity of particle trafficking and the speed of uncoating might also affect the status of the PIC when it reaches the nuclear membrane. Accelerated VSV-G induced trafficking through the cytoplasm, combined with a slower kinetics of uncoating due to CA mutations, may result in the shielding of TRN-SR2 nuclear localization signals when reaching the nuclear pore, forcing alternative import pathways to be selected. This could explain why the N74D mutant loses its dependency on TRN-SR2 in particular when combined with a VSV-G envelope.

Finally, CA may be directly involved in docking of PICs to the nuclear pore. Altered requirements of VSV-G pseudotyped HIV-1 Q63A/Q67A, E45A and N74D CA mutants for certain Nups, compared to wild type virus, were indeed demonstrated [32]. If the N74D mutation alters the selection of the specific Nup, alternative importins may be chosen for nuclear import, which could also explain the reduced TRN-SR2 dependency of the N74D CA mutant when carrying the HIV-1 envelope. The nature of these import factors still has to be revealed.

## Conclusion

Our data demonstrate that HIV-1 CA modulates nuclear entry of VSV-G pseudotyped viruses. The role of CA in nuclear import of viruses which retain their natural envelopes also warrants further study, since the N74D CA mutant is less dependent on TRN-SR2 for infection. Our results argue for careful interpretation of experiments performed with VSV-G pseudotyped viral particles when studying early steps in viral replication and the role of CA in uncoating, trafficking, docking at the NPCs and/or nuclear import. By playing a role in uncoating, trafficking and/or docking, steps that all precede the site-specific interaction between IN and TRN-SR2, CA may exert an indirect effect on the process of nuclear import. PIC nuclear import remains a bottleneck process in the viral life cycle and the interaction between HIV-1 IN and TRN-SR2 may provide an exciting new drug target [62]. In order to design drugs that can specifically interfere with the nuclear import of the PIC, a better understanding of the exact mechanisms of TRN-SR2-mediated nuclear import and possible other import pathways is required. The data presented here will aid in reaching this objective.

## Materials and methods

### Cell culture and siRNA transfections

HeLaP4 cells and 293T cells were grown in Dulbecco's modified Eagle's medium (DMEM) supplemented with 50 μg/ml gentamicin (Gibco, BRL) and 10% fetal calf serum (International Medical, Belgium). Cells were incubated at 37°C and 5% CO_2 _in a humidified atmosphere. Two different HeLaP4 cell lines stably knocked down for TRN-SR2 were generated by transduction with two different lentiviral vectors expressing shRNAs targeting the TRN-SR2 mRNA, called shTR3 and shTR4 (Sigma, clone-id NM_012470.2-867s21c1 and NM_012470.2-2084s21c1). A control cell line was established using a control vector expressing a scrambled shRNA referred to as shSCR (Sigma, product number SHC002). These lentiviral transfer plasmids were a kind gift from Dr. R. Hoeben (Leiden University Medical Center, The Netherlands). The shRNA expressing transfer plasmids are based on the pLKO.1 plasmid (Sigma) containing a puromycin resistance cassette. After transduction cells were selected and grown in DMEM as described above with the addition of 1 μg/ml puromycin.

For siRNA transfections, 6 × 10^5 ^HeLaP4 cells were plated per well in 6-well plates the day before transfection. The next day, 60 pmol of siRNA were transfected using 15 μl siLentFect (Bio-Rad, Belgium) in a total volume of 1 ml DMEM (10% serum, no antibiotics) per well according to the manufacturer's instructions. The siRNA siTRN-SR_2 targeting the TRN-SR2 mRNA (5' UCGGCGCACAGAAAUUAUAdTdT 3') (Qiagen, The Netherlands) was previously characterized [21]. As a control for non-specific effects, we used siTRN-SR_2MM targeting the same sequence but harboring 4 mutations (bold) (5' UCGGCGCA**GUCU**AAUUAUAdTdT 3') as described [21]. Mock transfected cells were always analyzed in parallel.

### Viruses and viral vectors

The viral molecular clones pNL4-3 and pNL4-3.Luc.R-E- were obtained through the NIH AIDS Research and Reagent Reference program, division of AIDS, NIAID, NIH, and were used to produce wild type HIV-1 virus and HIV-1 single-round luciferase reporter virus, respectively. The construct pNL4-3.Luc.R-E- N74D CA encoding a N74D CA mutant reporter virus was a kind gift of Dr. V. KewalRamani (National Cancer Institute, Maryland). Plasmid pNL4-3 N74D CA was created by digesting pNL4-3.Luc.R-E- N74D CA with BssHII and SpeI and cloning this fragment into pNL4-3 digested with BssHII and SpeI. Plasmids pNL4.3_LAI _cPPT and pNL4.3_LAI _cPPTD were used to produce wild type control viruses or viruses harboring an inactive DNA flap respectively as previously studied [38]. The following luciferase-encoding single-round reporter viruses were generous gifts from Dr. M. Emerman (Fred Hutchinson Cancer Research Center, Washington) [6,31]. Plasmid pLNCLuc is a MLV-derived retroviral transfer plasmid for production of MLV-based vector particles. Plasmid pLai3ΔenvLuc2 is an Env-deleted HIV-1 provirus and was the parental construct for the following HIV-based chimeric viruses (MHIV). The chimeric clone pMHIV-mMA12CA harbors the MLV MA, p12 and CA coding sequences instead of the HIV-1 MA and CA coding sequences [6]. In the chimeric clone pMHIV-mIN the HIV-1 IN coding sequence is replaced by the MLV IN coding sequence [31].

Viral vector production was performed as described earlier [63,64]. Briefly, VSV-G pseudotyped viral vectors were produced by triple polyethylenimine (PEI)-mediated transfection of 293T cells using per 10 cm dish 5 μg of the envelope expression plasmid pMD.G [65] for expression of VSV-G, 10 μg of a packaging plasmid and 20 μg of a transfer plasmid carrying a reporter gene flanked by 2 long terminal repeats. Two and three days after transfection, the supernatant was harvested, filtered with 0.45 μm pore-size syringe filters (Sartorius) and concentrated by centrifugal filtration using vivaspin concentrators (Sartorius). To produce viral vectors derived from HIV-1 we used pCHMWS_eGFP-T2A-Fluc [64] and pCMVΔR8.91 [66] as transfer and packaging plasmid respectively; for simian immunodeficiency virus (SIV) vectors we used pGAE-CAG-eGFP-Wpre [67] and pAd-SIV3+ [68] (a kind gift from Didier Nègre, Ecole Normale Supérieure, Lyon); for feline immunodeficiency virus (FIV) pSM32 and pSP26 [69] (a kind gift from Yea-Lih Lin, Institut de Génétique Humaine, Montpellier) were used; equine infectious anemia virus (EIAV) vectors were produced with p6.1G3CeGFPw (unpublished) and pEV53B [70] (kind gifts from M. Patel and J.C. Olsen, University of North Carolina, Chapel Hill) and for MLV-derived vectors we used pLNC_eGFP-T2A-Fluc and pCMVgagpol (Cell Biolabs, San Diego, CA). pLNC_eGFP-T2A-Fluc was constructed by cloning the eGFP-T2A-Fluc expression cassette from pCHMWS_eGFP-T2A-Fluc into the multiple cloning site in pLNCX (Clontech, Saint-Germain-en-Laye, France). Transfer plasmids pCSGFPW and pCASGFPW were used to produce lentiviral vectors harboring a DNA flap in sense or antisense orientation, respectively [37].

For production of Env-deleted pseudotyped single-round reporter viruses 20 μg of a viral molecular clone plasmid and 5 μg of an envelope expression plasmid were cotransfected per 10 cm Petri dish. Plasmids pMD.G [65] and pDOLHIVEnv [71] were used for pseudotyping viral particles with VSV-G or the HIV-1 envelope, respectively. Plasmid pAmpho [72] was a kind gift from Dr. M. Sena-Esteves (Massachusetts General Hospital, Boston) and was used for pseudotyping viral particles with the amphotropic MLV envelope. Plasmid pEboZΔ6GP (NTDL6) [73] was a kind gift from Dr. J. Wilson (Penn University, Philadelphia) and was used to pseudotype viruses with the Ebola Zaire (EboZ) envelope glycoprotein. Plasmids pCG-HcΔ18 and pCG-FcΔ30 [74] were a kind gift from Dr. C. Buchholz (Paul-Ehrlich-Institut, Langen), and were cotransfected (0.5 μg and 3.5 μg per Petri dish, respectively) during virus productions for pseudotyping with the measles virus envelope hemagglutinin and fusion protein, respectively. For productions of single-round viral particles pseudotyped by other envelopes than VSV-G, and for productions of the MHIV chimeric viruses, concentrated virus preps were used freshly and undiluted for infection because of the poor infectivity of these viral preparations. For production of wild type HIV-1 NL4-3 and NL4-3 N74D CA, the cells were transfected with 20 μg plasmid encoding the viral molecular clone. These viruses carry the wild type HIV-1 envelope; they were not concentrated but stored at -80°C after harvest and filtration through 0.45 μm pore-size syringe filters (Sartorius).

### Infection and transduction

1 × 10^4 ^HeLaP4 cells transiently or stably depleted of TRN-SR2 and control cells were seeded into 96-well plates. Two days later cells were infected in triplicate with 3 dilutions (typically 5 × 10^4^, 1 × 10^4 ^and 2 × 10^3 ^pg p24 unless described otherwise) of HIV-1 virus or VSV-G pseudotyped single-round HIV-1 virus in a total volume of 100 μl per well. p24 measurements were done with the HIV-1 p24 ELISA kit of PerkinElmer, or the INNOTEST HIV Antigen mAb kit of Innogenetics for the experiments presented in Figure 5. Virus stocks used in Figure 5 were also normalized for RT activity using the commercially available HS-Lenti RT Activity kit (Cavidi). We measured equal ratios of wild type and N74D mutant virus in both the pseudotyped and replication competent virus stocks using both the Innogenetics p24 ELISA and the RT activity kit. The inocula were replaced by fresh medium 24 h after infection. Each well was lysed 72 h after infection using 50 μl of lysis buffer (50 mM Tris/HCl, pH 7.3, 200 mM NaCl, 0.2% NP40, 5% glycerol) and was analyzed for β-galactosidase activity (chemiluminescent β-Gal Reporter Gene Assay, Roche Applied Science, Belgium) or firefly luciferase activity (ONE-Glo™, Promega, Belgium) according to the manufacturer's protocols. Chemiluminescence was measured with a Glomax luminometer (Promega, Belgium). The protein concentration of each sample was determined (BCA Protein Assay Kit, Thermo Scientific Pierce) and readouts were normalized to 1 μg total protein. The MHIV chimeric viruses and single-round viral particles pseudotyped with other envelopes than VSV-G were used freshly and undiluted for infection after concentration.

When retroviral vectors expressing GFP were used for transductions the inocula were adjusted to yield 30-60% GFP positive control cells. Transduction was performed 3 days post transfection of siRNAs and vectors were replaced by medium 24 h later. Three days post transduction, cells were trypsinized and fixed in 2% paraformaldehyde prior to analysis with a FACSCalibur flow cytometer (Becton-Dickinson, Franklin Lakes, NJ). Results are expressed as overall GFP fluorescence, i.e.% transduced cells × mean fluoresence intensity. For multiple-round infections of the stable TRN-SR2 knockdown HeLaP4 cells and control cells, 2 × 10^4 ^cells per well were plated the day before infection in a 6-well plate. The next day cells were infected with HIV-1 in a total volume of 1 ml corresponding to 6 × 10^4 ^pg p24 as determined by p24 measurements (HIV-1 p24 ELISA kit, PerkinElmer) of the viral stocks. Inocula were replaced by 4 ml of fresh medium containing only 5% fetal calf serum 24 h after infection. Four days after infection sampling of the supernatants for p24 analysis was started on a daily basis until cells reached complete confluency.

For the analysis of the late steps of viral replication, 5 × 10^5 ^HeLaP4 cells stably depleted of TRN-SR2 and control cells were seeded in a 6-well plate and grown until 50% confluency. Next 1 μg of pNL4-3 was transfected using 5 μl Fugene-6 according to the manufacturer's protocol. 6 hours after transfection cells were replenished with 1 ml fresh growth medium, and 24 hours after transfection supernatants were collected to measure p24 production (HIV-1 p24 ELISA kit, PerkinElmer). As a negative control, 5 μM of the protease inhibitor ritonavir was added to the shSCR expressing control cells.

### Recombinant protein purification

TRN-SR2 cDNA was amplified from pGEX-6P-1-TRN-SR2 (kind gift from Dr. W.-Y. Tarn, Institute of Biomedical Sciences, Taiwan) using a forward primer (5' AAAAGGATCCATGGAAGGAGCAAAGC 3') containing a BamHI site and a reverse primer (5' AAAACTCGAGCTACTATCGAAACAACCTGG 3') containing a XhoI site. The PCR fragment was digested with BamHI and XhoI and inserted into the pGEX-6P-2 plasmid digested with BamHI and XhoI. GST-TRN-SR2 was purified using Glutathione Sepharose Resin (GE Healthcare, Belgium) as described previously [21] with minor modifications. Plasmids pKBIN6H [75] and pETINH1 [76] were used for expression of recombinant C-terminally His_6_-tagged integrases derived from HIV-1 and MLV respectively. C-terminally His_6_-tagged integrases were purified by affinity and ion exchange chromatography as described [75]. The expression construct for His_6_-tagged human heterotrimeric G protein α oA subunit (His_6_-Gα_oA_) was kindly provided by Dr. D. Siderovski (University of North Carolina) and purified as described in [41]. His_6_-Roc-COR was purified via affinity and ion exchange chromatography as described in [42].

### AlphaScreen binding assay

The AlphaScreen binding assay (PerkinElmer) was performed in a total volume of 25 μl per well in 384-well Optiwell™ microtiter plates (PerkinElmer). Appropriate dilutions of recombinant GST-TRN-SR2 and IN-His_6 _were added to 5 μl of the assay buffer (25 mM Tris/HCl, pH 7.4, 150 mM NaCl, 1 mM MgCl_2_, 0.1% (v/v) Tween-20, 0.1% (w/v) BSA) to a total volume of 15 μl and the microtiter plates were incubated for 1 h at 4°C. Next, 10 μl of a mix of glutathione donor and nickel-chelate acceptor beads (PerkinElmer) (final concentration of 20 μg beads/ml) were added. Plates were then incubated for 1.5 h at room temperature and analyzed using an EnVision Multilabel Reader (PerkinElmer) according to the manufacturer's instructions. The assay was initially optimized for the interaction between GST-TRN-SR2 and HIV-1 IN-His_6 _by cross-titrations. A final concentration of 10 nM GST-TRN-SR2 was determined as optimal to avoid binding curve perturbation while still yielding a high signal-to-noise ratio (> 35). Subsequently, concentrations ranging from 1 to 150 nM of recombinant His_6_-tagged Gα_oA_, Roc-COR, HIV-1 IN or MLV IN proteins were used to titrate GST-TRN-SR2. Each titration was performed in duplicate and assays were independently repeated. The equilibrium dissociation constants (*K*_d_) were calculated with GraphPad Prism 5.

### Antibodies

For western blotting, protein concentrations of 1% SDS whole cell extracts were determined using the BCA protein assay (Thermo Scientific Pierce) and after separation by SDS-PAGE 30 μg of each extract was electroblotted onto polyvinylidene difluoride membranes. Membranes were probed with monoclonal antibodies against TRN-SR2 (1:100 dilution) (Genway biotech, USA), β-tubulin (1:10000 dilution) (Sigma) antibodies were used to confirm equal loading. Detection was performed using horseradish peroxidase-conjugated goat anti-mouse antibody (Dako) and chemiluminescence (ECL+, Amersham Biosciences). For FACS analysis of CD4 expression levels, 4 × 10^5 ^HeLaP4 or 293T cells were incubated with 5 μl R-phycoerythrin-conjugated anti-human CD4 antibody (Miltenyi Biotec) in a total volume of 150 μl PBS for 0.5 hour at 4°C. After incubation cells were washed with PBS, resuspended in 2% paraformaldehyde and analyzed by FACS.

### Quantitative PCR (QPCR)

To determine TRN-SR2 mRNA levels, RNA was extracted from 2 × 10^6 ^HeLaP4 cells using the Aurum total RNA Mini Kit (BioRad). 5 μg of total RNA was reverse transcribed to cDNA in 100 μl total volume using the High Capacity cDNA Reverse Transcription Kit (Applied Biosystems). cDNA corresponding to 25 ng of RNA was used to amplify TRN-SR2 mRNA or β-actin mRNA using the iQ Supermix (BioRad) and in-house designed primers and probes. Reactions and analysis were performed with the iQ5 Multicolor RT PCR Detection system and iQ5 Optical System software (BioRad). Each reaction contained 12.5 μl iQ Supermix, 5 μl cDNA, 100 nM or 200 nM forward primer, 100 nM or 200 nM reverse primer, 100 nM or 200 nM probe and water in a total volume of 25 μl for the detection of β-actin or TRN-SR2 mRNA, respectively. Primers and probe used for TRN-SR2 mRNA amplification and detection were: forward primer, 5'-CTACCAGATGTGGCTGAAGT-3'; reverse primer, 5'-ACAAAAAGTCGGTCTGTCAA-3'; probe, 5'-FAM-GCTCTGGGAGATCATGCAGG-TAMRA-3'. For β-actin mRNA detection we used: forward primer, 5'-CACTGAGCGAGGCTACAGCTT-3'; reverse primer, 5'-TTGATGTCGCGCACGATTT-3'; probe, 5'-HEX-ACCACCACGGCCGAGCGG-TAMRA-3'. Each sample was run in triplicate for 3 minutes at 95°C followed by 50 cycles of 10 seconds at 95°C and 30 seconds at 55°C.

## Competing interests

The authors declare that they have no competing interests.

## Authors' contributions

WT carried out the infection experiments, characterized the stable knockdown cells and wrote the manuscript. SDH generated the stable knockdown cell lines and carried out viral and vector experiments. JD performed the alphascreen protein-protein binding assays. OT, RV and MG purified the proteins used in the AlphaScreen assays. JDR helped with FACS analysis and drafting the manuscript. RG characterized and produced the different viral vectors used in this study. FC and ZD participated in the design and coordination of the study and wrote and edited the manuscript.

## Supplementary Material

Additional file 1 **Supplementary Figure 1. The DNA flap does not affect the TRN-SR2 dependency of HIV-1 replication**. (A) HeLaP4 cells depleted of TRN-SR2 (siTRN-SR_2) and control cells (mock and siTRN-SR_2MM) were challenged using 3 dilutions of an HIV-1-derived VSV-G pseudotyped viral vector harboring a functional wild type DNA flap (WT) or (B) with a flap-negative (Flap-) vector. Both vectors express GFP. Three days post transduction the overall GFP fluorescence in the cells was analyzed by FACS. Graphs show the mean values of GFP fluorescence ± SD of one representative experiment out of two performed in triplicate. (C) Same experiment as in (A), but using multiple-round HIV-1 NL4-3 (WT) or (D) HIV-1 NL4-3 flap-negative (Flap-) infectious viruses. Three days post infection β-gal activity was measured and normalized to the total amount of protein in the cell lysates. Graphs show the mean values of β-gal light units per μg protein (β-gal LU/μg protein) ± SD of one representative experiment out of two performed in triplicate. Click here for file
