# Supplementary figures and images for: Interplay between HIV Entry and Transportin-SR2 Dependency
Source: Retrovirology. 2011 Jan 30;8:7. doi: 10.1186/1742-4690-8-7 (PMC3041740; doi:10.1186/1742-4690-8-7)

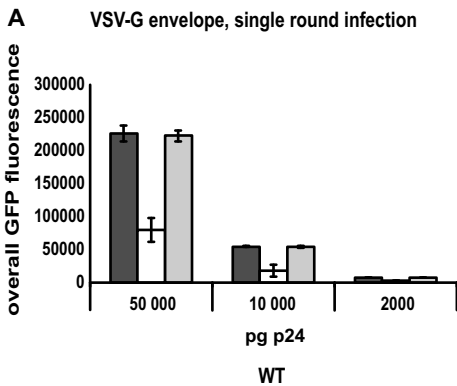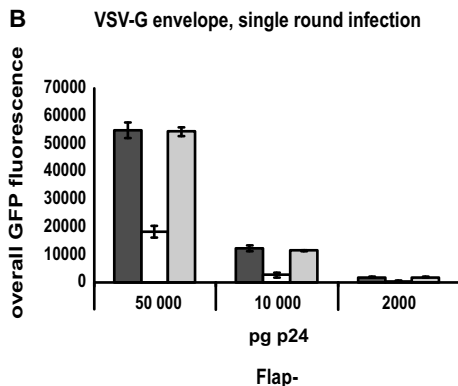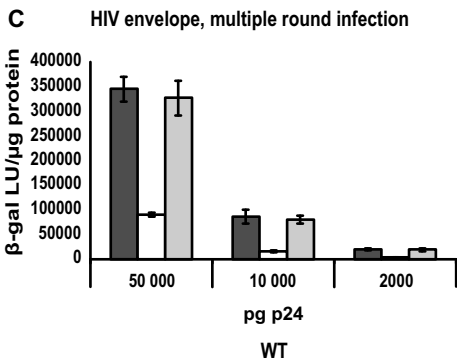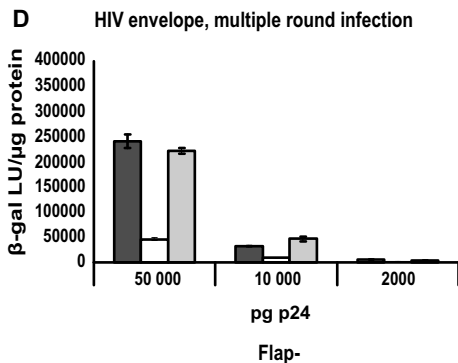

Supplement: Additional file 1 — Supplementary Figure 1. The DNA flap does not affect the TRN-SR2 dependency of HIV-1 replication. (A) HeLaP4 cells depleted of TRN-SR2 (siTRN-SR_2) and control cells (mock and siTRN-SR_2MM) were challenged using 3 dilutions of an HIV-1-derived VSV-G pseudotyped viral vector harboring a functional wild type DNA flap (WT) or (B) with a flap-negative (Flap-) vector. Both vectors express GFP. Three days post transduction the overall GFP fluorescence in the cells was analyzed by FACS. Graphs show the mean values of GFP fluorescence ± SD of one representative experiment out of two performed in triplicate. (C) Same experiment as in (A), but using multiple-round HIV-1 NL4-3 (WT) or (D) HIV-1 NL4-3 flap-negative (Flap-) infectious viruses. Three days post infection β-gal activity was measured and normalized to the total amount of protein in the cell lysates. Graphs show the mean values of β-gal light units per μg protein (β-gal LU/μg protein) ± SD of one representative experiment out of two performed in triplicate. [file 1742-4690-8-7-S1.PDF]
